# Study on the mechanism of tunable ferromagnetic composites with different rare earth ions

**DOI:** 10.1039/d1ra07249h

**Published:** 2021-11-18

**Authors:** Minli Zeng, Kunyapat Thummavichai, Wenting Chen, Guangsheng Liu, Zhen Li, Xiaorong Chen, Chen Feng, Yi Li, Nannan Wang, Yanqiu Zhu

**Affiliations:** Guangxi Institute Fullerene Technology (GIFT), Key Laboratory of New Processing Technology for Nonferrous Metals and Materials, Ministry of Education, School of Resources, Environment and Materials, Guangxi University Nanning 530004 China; College of Engineering, Department of Mathematics and Physical Sciences, University of Exeter Exeter EX4 4QF UK

## Abstract

Size-controlled Fe_3_O_4_ nanoparticles doped with rare earth (RE) ions (La^3+^, Ce^3+^, and Dy^3+^) varying from 15 nm to 30 nm were successful synthesized by a hydrothermal method for potential applications in the fields of biomedicine, environmental protection and magnetic memory devices. They possessed good dispersibility, adjustable particle size and nearly spherical shape. The particle grain size was uniformly distributed and showed a low degree of agglomeration in comparison with undoped Fe_3_O_4_ nanoparticles. The FTIR results showed that the RE elements partially replaced Fe^2+^, occupied the octahedral position, and enhanced the vibration of the Fe–O bond. The XPS study further revealed that the valence states of La, Ce, and Dy are both positive trivalent. The XPS Fe 2p valence band spectra observed a shift in the peak position toward higher binding energy after RE doping, confirming the existence of RE ions in the octahedral position. This paper explains the mechanism of rare earth doping with Fe_3_O_4_, and clarifies the influence of the doping of different RE ions on its magnetic properties. The detailed analysis of RE-doped ferrite materials can open a new perspective in designing biomedical and spintronics materials with tailored properties by choosing suitable cation substitution.

## Abstract 2

After doping RE elements, the corresponding *M*_s_ decreases, and the magnetization decreases with the increase of doping concentration. The RE ions were more inclined to replace some Fe^2+^ ions in the octahedral position from the VSM results.

## Graphical abstract

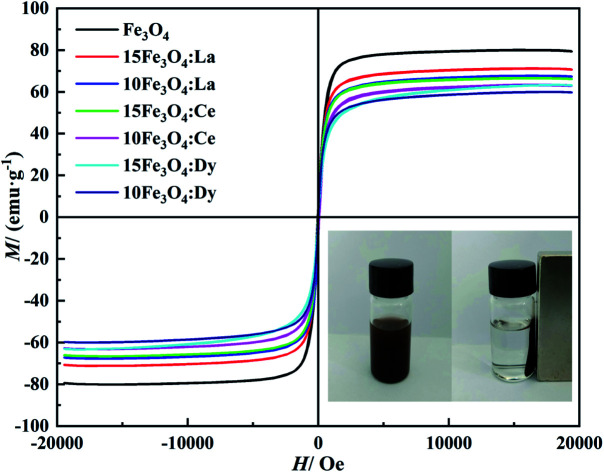

## 1. Introduction

Superparamagnetic Fe_3_O_4_ nanoparticles have attracted much attention for their potentials application in biomedicine, nanomedicine, environmental science and chemical production because of their chemical stability, innocuousness, high saturation magnetization (*M*_s_), and inexpensiveness.^1–4^ The magnetic properties of nanoparticles are strongly influenced by their size distribution, chemical composition and magnetic interaction. It is worth noting that on the nanometer scale, due to the surface spin effect, the decrease of particle size usually means the reduction of saturation magnetization. In this case, Fe_3_O_4_ nanoparticle clusters represent a promising system with high *M*_s_, size control and magnetism control by rare earth (RE) doping.^5–7^

RE elements have a special electronic structure with 4f orbitals buried inside the atom and shielded by 5s and 5p electrons, making the 4f electrons in the inner layer unaffected by the surrounding environment. The orbital contribution to the magnetic moment is not suppressed by the electrostatic field of other surrounding ligand atoms. Therefore, when calculating magnetic moment, we should consider the contribution of both spin and orbit. RE magnetism depends on the number of unpaired 4f electrons, and hence, their optical and electronic phenomena have been applied to introduce magnetic field effects.^8,9^ The 4f orbital of RE ions are in an unfilled state, which can be filled with electrons, offering broad research in magnetic and optical fields.^10,11^ By reducing the size of materials to the nanoscale, it can help promote many special properties of the materials, due to the small size effect, quantum size effect, surface effect and macroscopic quantum tunnel effect. When the size of the magnetic material reaches about 100 nm, the magnetic material will become a single domain particle, *i.e.*, a particle that contains only one single magnetic domain.^12,13^

Relevant research shows that the physical and chemical properties of Fe_3_O_4_ can be affected by doping modification. Lastovina *et al.* prepared Sm-doped magnetic Fe_3_O_4_ nanoparticles by a solvothermal polyol method. By introducing 2,2′-bipyridine in the synthesis process, the average particle size was reduced to about 9 nm. The *M*_s_ of Sm-doped MNPs and MNPs-Bpy samples were 71.6 and 68.8 emu g^−1^, respectively.^14^ Paransa prepared Nd–Ce doped Fe_3_O_4_-chitosan nanocomposites with a core–shell structure by coprecipitation and cross-linking method. The results showed that the magnetic response and catalytic activity were improved after doping Nd^3+^ and Ce^3+^.^15^ Andrade Neto *et al.* prepared Fe_3_O_4_ doped with Ce^4+^, Co^2+^, Mn^2+^ and Ni^2+^ by coprecipitation method. The research presented that the grain size decreased after doping, and the intermediate energy level was generated near the conductive band, which increased the energy gap of the Fe_3_O_4_ nanoparticles and enhanced their photocatalytic performance. Doping defects increased *M*_s_, but decreased residual magnetization (*M*_r_). The addition of cobalt reduced the resistivity of Fe_3_O_4_, while the addition of Ce, Mn and Ni increased the resistivity of Fe_3_O_4_.^16^

Many studies have reported that RE elements replace Fe^3+^ or Fe^2+^ in Fe_3_O_4_ crystals to influence their physical and magnetic properties.^17–22^ However, relatively little attention has been paid to the effects of different RE elements doping and the doping amount on the structure of magnetite Fe_3_O_4_ nanoparticles. In this paper, Fe_3_O_4_ nanoparticles doped with various RE elements, *i.e.*, La^3+^, Ce^3+^ and Dy^3+^, were synthesized *via* hydrothermal technique. The morphologies and structure of each doping sample will be investigated. The size control of magnetite Fe_3_O_4_ nanoparticles by RE doping and their magnetic properties will be reported, and promoted a new direction for new medical materials and magnetic memory devices. Not only that, but this research will also be beneficial to environmental protection (such as adsorption of industrial and urban wastewater, and degradation of organic polluted water).^23,24^

## 2. Experimental details

### 2.1. Materials and reagents

All of the chemicals are commercially available and used as received without further purification. They include ferric chloride hexahydrate (FeCl_3_·6H_2_O, GHTECH), cerium chloride heptahydrate (CeCl_3_·7H_2_O, GHTECH), lanthanum chloride hexahydrate (LaCl_3_·6H_2_O, GHTECH), dysprosium nitrate hexahydrate (DyNO_3_·6H_2_O, Aladdin), ethylene glycol ((CH_2_OH)_2_, GHTECH), polyethylene glycol (HO(CH_2_CH_2_O)_*n*_OH, Macklin, average molar weight = 6000), sodium acetate (CH_3_COONa or NaAc, GHTECH). FeCl_3_·6H_2_O and RE compounds (CeCl_3_·7H_2_O, LaCl_3_·6H_2_O, DyNO_3_·6H_2_O) were selected as synthetic raw materials. (CH_2_OH)_2_ was used as a reducing agent and surfactant. CH_3_COONa and HO(CH_2_CH_2_O)_n_OH were used as auxiliary precipitation reagents. The water used in the whole research process was deionized water.

### 2.2. Synthesis of rare earth-doped Fe_3_O_4_ nanoparticles

5.0 mmol FeCl_3_·6H_2_O and a certain amount of RE compounds (CeCl_3_·7H_2_O, LaCl_3_·6H_2_O, DyNO_3_·6H_2_O) were dissolved and mixed in 80 mL of ethylene glycol according to the ratios of 10 : 1 and 15 : 1, respectively, and then 7.2 g of sodium acetate and 2.0 g of polyethylene glycol were added. The mixture was mechanical stirred in a water bath at 70 °C for 1.0 h, and further sealed in a 100 mL stainless steel autoclave lined with Teflon. After that, the sealed autoclave was heated and maintained at 200 °C for 12.0 h, then naturally cooled down to room temperature to form a black precipitate. The obtained black precipitate was washed several times with ethanol and deionized water. The black particles were collected by centrifugation and dried overnight at 60 °C in an oven to obtain RE (La^3+^, Ce^3+^, Dy^3+^) doped Fe_3_O_4_ particles. The ratios of Fe to RE elements of 10 : 1 and 15 : 1 are respectively marked as 10Fe_3_O_4_:RE and 15Fe_3_O_4_:RE (such as 10Fe_3_O_4_:La and 15Fe_3_O_4_:La, RE represents La^3+^, Ce^3+^, Dy^3+^, respectively).

### 2.3. Characterization

An X-ray diffractometer (XRD, Rigaku D/MAX 2500 V) was used to analyze the crystal phase of the as-prepared products. The wavelength of the copper target was *λ* = 1.5406, the working current and accelerating voltage were 100 mA and 40 kV, respectively. The average crystallite size of all samples was estimated by the Scherrer equation.^25^ The morphology and nanostructure of the samples were analyzed by transmission electron microscope (TEM, Tecnai F20, operated at 200 kV). In the range of 400–4000 cm^−1^, the chemical bonds in the samples were studied by Fourier transform infrared spectroscopy (FTIR, Spectrum Two, PerkinElmer). The material was characterized by vibrating sample magnetometer (VSM, PARC 155) at room temperature with a maximum applied magnetic field of 2 T. X-ray photoelectron spectroscopy (XPS, ESCALAB 250Xi) was used to analyze the chemical properties of the material surface, the surface element composition, and valence state of the material. Binding energies of all elements were corrected in reference to the C 1s peak at 284.6 eV.

## 3. Results and discussion

### 3.1. Structural and morphology analysis

#### 3.1.1. XRD analysis

**Fig. 1:**
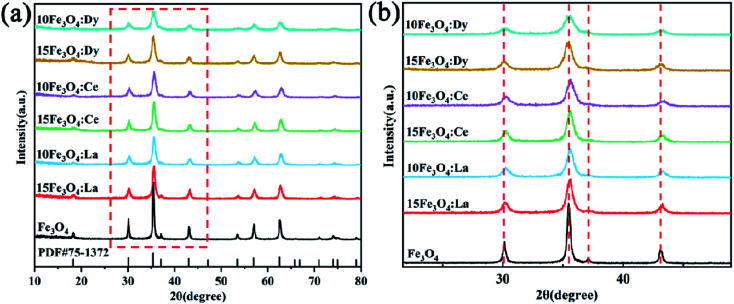
(a) XRD patterns of RE-doped Fe_3_O_4_; (b) enlarged view of the XRD patterns in (a).

Fig. 1 is the XRD pattern of the all as-prepared samples. It can be seen from the pattern that the position of the diffraction peak appearing in the Fe_3_O_4_ test curve corresponds to the face-centered cubic magnetite structure (JCPDS no. 75-1372), indicating that the Fe_3_O_4_ crystal has a good inverse spinel structure and the diffraction peak shape is sharp. The particle size distribution is narrow, which proves the successful preparation of the Fe_3_O_4_ nanoparticles. Peaks are observed at 2*θ* = 30.27°, 35.74°, 37.09°, 43.47°, 57.19°, and 62.73°, corresponding to the Bragg reflections of the (220), (311), (222), (400), (511), and (440) planes, respectively. From Fig. 1(a), we also found that the XRD pattern of the RE-doped Fe_3_O_4_ sample is wider than that of pristine Fe_3_O_4_, indicating that doping RE elements does not change the original inverse spinel structure of Fe_3_O_4_, while it does affect the particle size.^26^ Moreover, the XRD patterns of the La^3+^- and Ce^3+^-doped nanoparticles shifted to a higher angle compared with pristine Fe_3_O_4_ (Fig. 1(b)), which is probably due to La^3+^ and Ce^3+^ (ionic radius of 106.1 pm and 103.4 pm, respectively) replacing either Fe^2+^ (61.78 pm) or O atoms (140 pm),^19^ resulting in smaller lattice parameters and smaller interplanar spacing. No obvious shifting of peaks can be detected in the Dy^3+^-doped sample, which is probably because the ionic radius of Dy^3+^ (91.2 pm) is closer to the size of Fe^2+^ (61.78 pm).^27^ Hence, there are more substituted Fe atoms and fewer substituted O atoms. The peak position presents no obvious changes, while the diffraction peaks become broader as the amount of doping increases. An observed broad XRD peak can lead to the decrease of grain size and the increase of the lattice stress caused by the existence of RE atoms in the Fe_3_O_4_ lattice.^7^

The average crystallite diameter of the samples was calculated from the strongest peaks based on the Debye–Scherrer formula:

1 *D* = *kλ*/*β* cos *θ*

where *D* is the crystallite size in Å, *λ* is the wavelength in Å, *β* is the FWHM in radian and *θ* is the scattering angle in degree, *k* = 0.89.

As can be seen from Table 1, the average particle size of the doped nanoparticles is about 27 nm, which is half smaller than that of pristine Fe_3_O_4_ (56 nm). The size increases with the increase of doping concentration, which may be that RE ion (La^3+^, Ce^3+^, Dy^3+^) doping inhibits the growth of Fe_3_O_4_ grains. The concentration of oxygen vacancies (V_O_) in the material is caused by the continuous decrease of RE doping. Grain growth is affected by grain boundary movement, which is mainly affected by the diffusion of V_O_.^28^ Therefore, the existence of V_O_ was conducive to ion diffusion, promotes grain growth, reduced the concentration of V_O_, reduced the rate of grain boundary movement, slowed down grain growth and inhibited grain growth. From defect chemistry, the concentration of iron vacancies (V_Fe_) increased when RE ions (La^3+^, Ce^3+^, Dy^3+^) are doped into ferrite. Thus, the concentration of V_O_ decreased, slowing down the movement rate of grain boundaries, and thus inhibiting grain growth.^29^ In addition, after RE doping, some of the RE ions (La^3+^, Ce^3+^, Dy^3+^) could not enter the crystal lattice, although they were distributed at or near the grain boundary, which increased the stress of the grain boundary, hindered the movement of the grain boundary and inhibited the growth of the grain.

**Table 1:** Effect of rare earth doping on grain size

Samples	Fe_3_O_4_	15Fe_3_O_4_:La	10Fe_3_O_4_:La	15Fe_3_O_4_:Ce	10Fe_3_O_4_:Ce	15Fe_3_O_4_:Dy	10Fe_3_O_4_:Dy
Crystallite size/nm	55.91	29.69	28.92	28.88	25.73	27.86	23.12

#### 3.1.2. SEM analysis

**Fig. 2:**
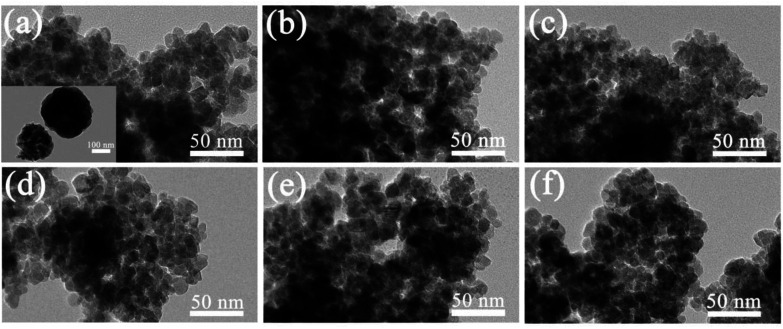
TEM images of (a) 15Fe_3_O_4_:La, (d) 10Fe_3_O_4_:La, (b) 15Fe_3_O_4_:Ce, (e) 10Fe_3_O_4_:Ce, (c) 15Fe_3_O_4_:Dy, and (f) 10Fe_3_O_4_:Dy nanoparticles. The inset in (a) shows the TEM image of pristine Fe_3_O_4_ nanoparticles.

The morphology analysis of the RE-doped Fe_3_O_4_ system was confirmed by TEM images (Fig. 2(a–f)). As shown in the inset of Fig. 2(a), pristine Fe_3_O_4_ is uniformly dispersed and spherical, uniform in morphology, large in size, about 200 nm in size, and serious in particle agglomeration. The TEM images show that RE doping greatly reduced the particle size to about 20 nm, which is in good agreement with the XRD results, and no round clusters are formed among the particles (Fig. 2(a–f)). It also shows that with the increase of the doping amount, the degree of sample agglomeration decreases.

#### 3.1.3. FTIR analysis

**Fig. 3:**
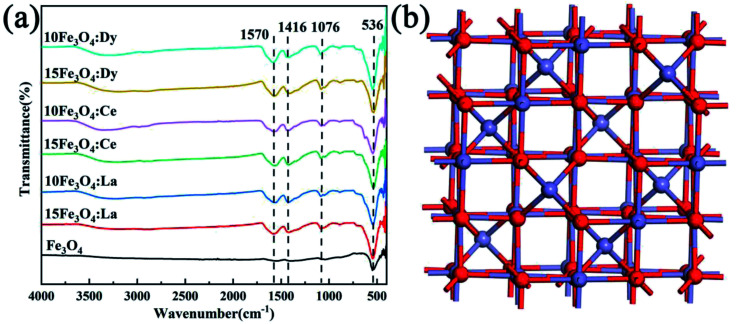
(a) The infrared spectrum of the sample, (b) the ball and stick model of Fe_3_O_4_, where red represents O atoms and blue represents Fe atoms.

Fig. 3(a) shows the infrared spectrum of all samples. The characteristic peak of Fe_3_O_4_ appears at 534 cm^−1^, which is attributed to the tensile vibration of the Fe–O bond. A weak peak at 434 cm^−1^ is probably due to the existence of the Fe^3+^–O^2−^ bond at the octahedral position, which confirmed the formation of spinel Fe_3_O_4_. The broad, weak band found at about 3446 cm^−1^ was attributed to the stretching and bending of the hydroxyl vibration of absorbed water molecules on the surface of Fe_3_O_4_. The 3446 cm^−1^ peak gets narrower and sharper after doping, but the peak becomes narrower and the peak intensity becomes larger after doping, which indicates that there is superposition of the OH stretching band in ethylene glycol and the degree of water absorption increases.^30^ The stretching vibration of the carbonyl group has a strong infrared absorption peak at 1564, 1410 and 1076 cm^−1^, which can also be related to CH_3_COONa.^31^ Compared with pristine Fe_3_O_4_, there is no significant shift in peak position of the infrared spectrum in the RE-doped Fe_3_O_4_. However, the transmittance of the characteristic peak is obviously enhanced, which proves that the RE elements were successfully doped into the Fe_3_O_4_ lattice, partially replaced Fe^2+^, occupied the octahedral position, and enhanced the vibration of the Fe–O bond.^19^Fig. 3(b) is a ball stick model diagram of Fe_3_O_4_, in which red represents the O atom and blue represents the Fe atom, in which Fe^2+^ occupies half of the octahedral voids, and Fe^3+^ occupies the remaining half of the octahedral voids and all tetrahedral voids.

#### 3.1.4. XPS analysis

**Fig. 4:**
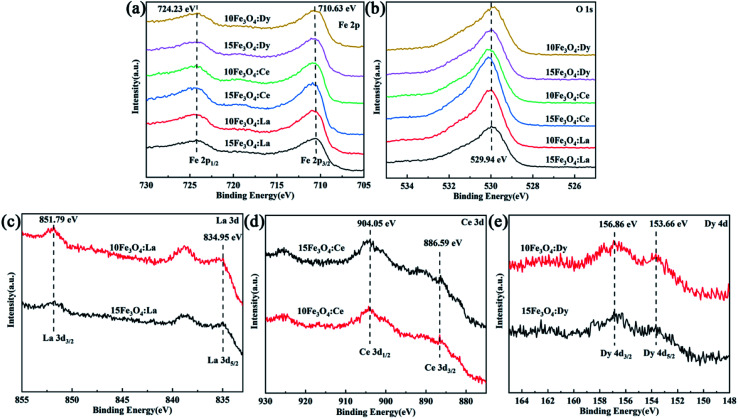
XPS spectra of the nanoparticles: (a) Fe 2p spectra of each sample, (b) O 1s spectra of each sample, (c) La 3d spectra of the La^3+^-doped nanoparticles, (d) Ce 3d spectra of the Ce^3+^-doped nanoparticles, and Dy 4d spectra of the Dy^3+^-doped nanoparticles.

In order to study the surface chemical properties and valence states of the RE-doped nanoparticles, XPS analysis was carried out on the samples. Fig. 4(a) shows the XPS spectrum of Fe 2p. The peaks at 724.23 eV and 710.63 eV correspond to Fe 2p_1/2_ and Fe 2p_3/2_, respectively, which are mainly attributed to the Fe–O bond and are very close to the reported data for Fe_3_O_4_ in the literature (724.63 eV and 710.97 eV, respectively).^20^ Compared with the literature, with the increase of the doping amount, the Fe 2p_1/2_ and Fe 2p_3/2_ of the doped samples shift towards low binding energy, which may be due to the RE ions replacing Fe^2+^ and changing the ratio of Fe^3+^:Fe^2+^.^22^ The observation of the peak shifting in Fe 2p_3/2_ of the La^3+^ and Ce^3+^-doped samples being larger than that of the Dy^3+^-doped samples could be due to the fact that the ionic radius of Dy^3+^ is closer to Fe^2+^, resulting in a small change of the Fe 2p_3/2_ peak position. Satellite peak appears near 718 eV, which proves the successful doping of RE elements in Fe_3_O_4_.^32,33^ In Fig. 4(b), the characteristic peak at 529.94 eV corresponding to the O 1s orbital in Fe_3_O_4_ was observed, which is in agreement with the previous reported results in the literature (530.0 eV).^34^ Among them, O 1s of the Dy^3+^-doped sample shifts toward a lower binding energy, while the La^3+^- and Ce^3+^-doped samples shift toward higher binding energy. We considered that the doping elements substitution of V_O_ by doping elements, which is consistent with XRD pattern analysis. The XPS spectrum of La 3d, the peaks are observed at 851.79 eV and 834.95 eV correspond to La 3d_3/2_ and La 3d_5/2_, respectively (Fig. 4(c)), indicating that La^3+^ is doped and La exists in the La^3+^ valence state.^35^ XPS spectrum of Ce 3d in Fig. 4(d) confirms the successful incorporation of Ce ions in the Fe_3_O_4_ lattice. The two main peaks at 904.05 eV and 886.59 eV were assigned to Ce 3d_1/2_ and Ce 3d_3/2_.^20^Fig. 4(e) shows the XPS spectrum of Dy 4d. The peaks at 156.86 eV and 153.66 eV correspond to Dy 4d_3/2_ and Dy 4d_5/2_, respectively, indicating the successful incorporation of Dy inside the Fe_3_O_4_ structure framework with the main chemical state of Dy^3+^.^36,37^ It is clear from Fig. 4(a and b) that RE doping would affect the surface properties of the original Fe_3_O_4_. However, the increase of the doping amount does not change its surface chemical state (Fig. 4(c–e)), indicating that the doping amounts are within a reasonable range and had little effect on its surface crystal properties.

The atomic concentration values of each element from each sample are summarized in Table 2. The results show that the RE/Fe ratios of 15Fe_3_O_4_:RE are all smaller than that of 10Fe_3_O_4_:RE, which is consistent with the theoretical results. The atomic ratios of La/Fe of 15Fe_3_O_4_:La and 10Fe_3_O_4_:La are shown below 0.1 (the theoretical values are 0.15 and 0.1, respectively), indicating that the doping amount of La is less. The Ce/Fe atomic ratios of 15Fe_3_O_4_:Ce and 10Fe_3_O_4_:Ce are 0.12 and 0.15, respectively, which are close to the theoretical values. The Dy/Fe atomic ratios of 15Fe_3_O_4_:Dy and 10Fe_3_O_4_:Dy are 0.10 and 0.15, respectively, which are consistent with the theoretical values.

**Table 2:** XPS atomic ratios of all samples

	O (at%)	Fe (at%)	La (at%)	Ce (at%)	Dy (at%)
15Fe_3_O_4_:La	66.32	32.34	1.35	—	—
10Fe_3_O_4_:La	67.67	30.66	1.67	—	—
15Fe_3_O_4_:Ce	64.41	31.71	—	3.88	—
10Fe_3_O_4_:Ce	65.51	30.01	—	4.48	—
15Fe_3_O_4_:Dy	65.30	31.49	—	—	3.21
10Fe_3_O_4_:Dy	65.41	29.93	—	—	4.66

### 3.2. Magnetic performance analysis

**Fig. 5:**
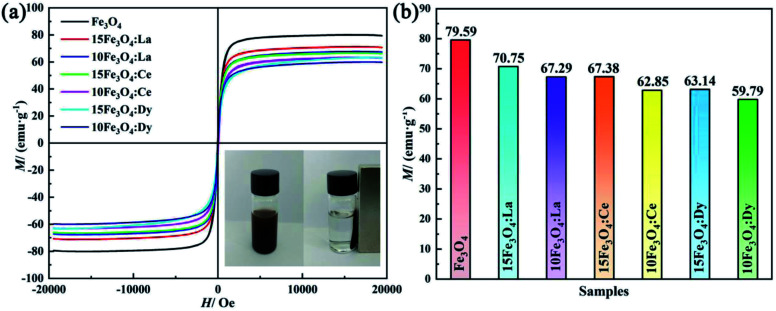
(a) The magnetization curves of the nanoparticles at room temperature; the inset in (a) is a picture of Fe_3_O_4_ before and after separation with a magnet; (b) the magnetization of the different nanomaterials.

In order to study the magnetic properties of the samples, VSM analysis was carried out. Fig. 5(a) shows the hysteresis curve of the corresponding samples, and all particles are superparamagnetic. The *M*_s_ of magnetic Fe_3_O_4_, 15Fe_3_O_4_:La, 10Fe_3_O_4_:La, 10Fe_3_O_4_:Ce, 10Fe_3_O_4_:Ce, 15Fe_3_O_4_:Dy and 10Fe_3_O_4_:Dy are about 79.59, 70.75, 67.29, 67.38, 62.85, 63.14 and 59.79 emu g^−1^, respectively (as shown in Fig. 5(b)). The results indicate that the *M*_s_ of pristine Fe_3_O_4_ is lower than that of standard block Fe_3_O_4_ (96.43 emu g^−1^).^19^ After doping RE elements, the corresponding *M*_s_ decreases significantly, and the magnetization decreases with the increase of doping concentration. Compared with the pristine Fe_3_O_4_ nanoparticles (*M*_s_ value of 79.59 emu g^−1^), the La^3+^-doped sample shows the lowest decrease (11.1%) in the *M*_s_, whereas the highest decrease (24.9%) is found for the Dy^3+^-doped sample. As for nanoparticles, their ultrafine particle properties, surface disorder and cation distribution are the main reasons for the decrease of *M*_s_. The decrease of *M*_s_ is due to the lack of oxygen-mediated super exchange mechanism between the surface iron ions, which leads to the decrease of the exchange coupling and the inclined spin of the surface layer, so there will be a magnetic dead surface layer.^38,39^ Another reason may be that Fe_3_O_4_ can easily absorb water after doping (as shown by infrared spectrum), thus reducing the volume fraction of the magnetic materials. The magnetic moment on the octahedral position of Fe_3_O_4_ is antiferromagnetic. Meanwhile, the magnetic moment on the tetrahedral position is ferromagnetic. Moreover, the magnetic moment of the unit cell only comes from the Fe^2+^ ions. XRD and FTIR data suggested that RE ions were more inclined to the octahedral position and replaced some Fe^2+^ ions. These RE ions are nonmagnetic (without unpaired d electrons), and they would replace some magnetic Fe^2+^ ions in the octahedral position.^19^ Hence, this reduces the magnetic moment of the nanomaterials, as expected. The main reason for the different *M*_s_ value of the RE-doped Fe_3_O_4_ samples is perhaps related to the difference of the ionic radius of La^3+^, Ce^3+^ and Dy^3+^. The ionic radius of Dy^3+^ and Fe^2+^ are relatively close, so the number of replacements for Fe^2+^ is greater. This results in a decrease in the magnetic moment of the unit cell and *M*_s_, which agrees with the results obtained from the XRD analysis mentioned above. However, the radii of the La^3+^ and Ce^3+^ ions are quite different compared with that of Fe^2+^ ions. The number of substituted Fe^2+^ ions is not as large as that of Dy^3+^. The magnetic moment in the crystal thus decreases less, causing higher *M*_s_ level than that of the Dy^3+^-doped nanoparticles. The radius of La^3+^ (106.1 pm) is slightly larger than that of Ce^3+^(103.4 pm). As a result, the *M*_s_ of La^3+^ is greater than the *M*_s_ of the Ce^3+^-doped samples. Therefore, the overall magnetization degree can be summarized as follows: *M*_s_ (Fe_3_O_4_) > *M*_s_ (15Fe_3_O_4_:La) > *M*_s_ (15Fe_3_O_4_:Ce) > *M*_s_ (15Fe_3_O_4_:Dy), *M*_s_ (Fe_3_O_4_) > *M*_s_ (10Fe_3_O_4_:La) > *M*_s_ (10Fe_3_O_4_:Ce) > *M*_s_ (10Fe_3_O_4_:Dy). It can be observed from Fig. 5(a) that the *M*_s_ of the nanoparticles decreases with the increase of doping amount. Furthermore, the number of substituted Fe^2+^ increases due to the increase of doping amount, which leads to the decrease of *M*_s_ and is also consistent with XRD analysis. In addition, the inset image of Fig. 5(a) clearly shows that Fe_3_O_4_ nanoparticles can be completely separated in water by providing an external magnetic field, showing strong magnetism and good dispersibility.

## 4. Conclusion

Pristine Fe_3_O_4_, 15Fe_3_O_4_:La, 10Fe_3_O_4_:La, 15Fe_3_O_4_:Ce, 10Fe_3_O_4_:Ce, 15Fe_3_O_4_:Dy, and 10Fe_3_O_4_:Dy nanomaterials with superparamagnetic properties were synthesized by hydrothermal method with uniform particle size distribution and dispersibility. XRD data confirmed that the sizes of Fe_3_O_4_ were adjusted by doping. RE elements doping can inhibit crystal growth and reduce the particle size of Fe_3_O_4_. With the increase of the doping amount, the particle sizes decreased accordingly. The XRD pattern of the doped nanoparticles shifted to a high angle, indicating that part of the doped atoms replaced O atoms, resulting in a smaller lattice parameter and a smaller interplanar spacing. XRD and XPS analyses confirmed the successful doping of RE elements into the framework of Fe_3_O_4_. The chemical states of La, Ce, and Dy are positive trivalent. The XPS spectrum of Fe 2p doped with RE shifts to the direction of high binding energy. It demonstrated that RE ions existed in the octahedral position, and the increase of RE doping had a slight effect on the surface properties of the nanoparticles. The RE ions were more inclined to replace some Fe^2+^ ions in the octahedral position from the VSM results. All of the nanoparticles were superparamagnetic. The doping of La^3+^, Ce^3+^ and Dy^3+^ reduced the *M*_s_ of Fe_3_O_4_, and *M*_s_ decreased with the increase of the doping amount. The results of the magnetization curve can be summarized as *M*_s_ (Fe_3_O_4_) > *M*_s_ (15Fe_3_O_4_:La) > *M*_s_ (15Fe_3_O_4_:Ce) > *M*_s_ (15Fe_3_O_4_:Dy), *M*_s_ (Fe_3_O_4_) > *M*_s_ (10Fe_3_O_4_:La) > *M*_s_ (10Fe_3_O_4_:Ce) > *M*_s_ (10Fe_3_O_4_:Dy). These materials have potential applications in magnetic storage devices, biomedical applications and environmental protection, such as the adsorption of industrial wastewater and the degradation of organic polluted wastewater.

## Conflicts of interest

There are no conflicts to declare.

## Supplementary Material
